# Comparing the efficacy and safety of safinamide with rasagiline in China Parkinson’s disease patients with a matching adjusted indirect comparison

**DOI:** 10.1038/s41598-025-94960-9

**Published:** 2025-03-26

**Authors:** Yuyan Tan, Qianqian Wei, Pingyi Xu, Enxiang Tao, Lijiao Wang, Carlo Cattaneo, Huifang Shang, Shengdi Chen

**Affiliations:** 1 Department of Neurology, Ruijin Hospital, Shanghai Jiao Tong University School of Medicine, Shanghai, China; 2 Department of Neurology, West China Hospital of Sichuan University, Chengdu, China; 3 Department of Neurology, The First Affiliated Hospital of Guangzhou Medical University, Guangzhou, China; 4 Department of Neurology, Sun Yat-sen Memorial Hospital, Guangzhou, China; 5 Medical and Regulatory Department, Hainan Zambon Pharmaceutical Co., Ltd, Shanghai, China; 6 Medical Department, Zambon SpA, Bresso, Italy

**Keywords:** Safinamide, Rasagiline, Levodopa, Monoamine oxidase type B inhibitors, Matching-adjusted indirect comparison, Parkinson’s disease, Parkinson's disease, Pharmacodynamics

## Abstract

Safinamide and rasagiline are adjuncts to levodopa for the motor fluctuations of Parkinson’s Disease (PD). However, there remains a scarcity of head-to-head studies that directly compare safinamide and rasagiline. This study compared safinamide and rasagiline as adjuncts to levodopa in Chinese PD patients with motor fluctuations by matching-adjusted indirect comparison. Baseline age, sex, BMI, and OFF time were adjusted for matching. Efficacy outcomes were the mean changes in total daily OFF time, UPDRS III, and PDQ-39 from baseline to week 16, which calculated by a weighted covariance model. Safety outcomes included rates of AEs, SAEs, and DCAEs. Bucher method was used for mean difference (MD) of efficacy and odds ratio (OR) of safety outcomes. Combination therapy of safinamide 50-100 mg/day and levodopa significantly reduced the mean total daily OFF time by 0.7 h (− 1.40 to − 0.02) compared to the combination therapy of rasagiline 1 mg/day and levodopa. Safinamide more effectively reduced UPDRS III (− 2.9, − 5.28 to − 0.52). Changes in PDQ-39 scores indicated a trend toward greater improvement in safinamide. There was no significant difference in safety outcomes. Compared to rasagiline, the combined therapy of safinamide and levodopa could significantly improve motor fluctuations for PD patients in China, without compromising safety.

## Introduction

Parkinson’s disease (PD) is a common progressive neurodegenerative disorder characterized by motor symptoms, including rigidity, resting tremor, bradykinesia, and postural instability^1^. Levodopa has been used for PD motor symptoms since 1967 and remains the gold standard treatment^2,3^. However, after levodopa treatment, 50% of patients diagnosed with PD will experience motor fluctuations within three to five years of disease onset^4^. By ten years after the onset, up to 80% of PD patients are affected by motor fluctuations, which become one of their most troublesome symptoms^4^. Therefore, the use of levodopa adjunctive drugs is necessary^4^. Monoamine oxidase type B inhibitors (MAO-BIs) are adjunctive drugs for levodopa, whose efficacy and safety have been demonstrated in both clinical trials and pooled analysis^5^. MAO-BIs have been recommended as the adjunctive drugs to levodopa by multiple definitive guidelines both in China and overseas^6–8^.

As one of the MAO-BIs, rasagiline could increase the dopamine in the brain by blocking MAO-B related to dopamine metabolism. Safinamide is a dual-action agent, serving as both an MAO-BI and a channel blocker. It not only enhances dopamine levels in the brain, but also could block the voltage-gated Na^+^ and Ca^2+^ channels and reduce the glutamate release^5,9,10^. Safinamide may differ from rasagiline in efficacy and safety due to the different mechanisms. A retrospective study showed significant symptom relief in 52.9% PD patients with symptoms of wearing-off 4–6 months after switching from 1 mg rasagiline to 100 mg safinamide, indicating the potential benefit of safinamide^11^. There remains a scarcity of head-to-head studies that directly compare safinamide and rasagiline. However, there was a network meta-analysis study included 31 randomized controlled trials published from 1983 to March 2022 on MAO-BI plus and MAO-BIs, encompassed a global population. In this study, the safety and efficacy of safinamide and rasagiline were indirectly compared by researchers, and the result showed there was no significant difference in improving PD patients’ efficacy and safety between rasagiline and safinamide^5^. The study did not perform additional subgroup analyses to evaluate efficacy across different populations, especially with respect to the Chinese population. The absence of such analyses is likely due to the insufficient availability of literature data specific to the Chinese demographic during the time period of this meta-analysis^12^. Given that China has the largest number of PD patients globally, it is essential to investigate the differences in efficacy and safety between safinamide and rasagiline in improving Parkinson’s symptoms within the Chinese population. Such research could provide valuable data to advance the field.

This study used an anchored MAIC method to compare safinamide and rasagiline as adjunctive treatment to levodopa in Chinese PD patients suffering from motor symptoms. This might be beneficial to clinical decision-making in selecting the optimal treatment to maintain the levodopa effect.

## Methods

### Systemic literature review

PubMed, Embase, Web of Science, and Cochrane were searched from inception to December 13, 2023. Eligibility criteria include (i) eligible randomized controlled trials (RCTs); (ii) participants were Chinese patients diagnosed with PD (iii) studies comparing drugs of interest (rasagiline and safinamide) with placebo or with each other, adjunctive to levodopa. Three RCTs of safinamide (*n* = 1)^13^ and rasagiline (*n* = 2)^14,15^ in Chinese PD patients were found.

XINDI study (NCT03881371) was a phase III, multicenter, randomized, double-blind, placebo-controlled clinical trial, which evaluated the efficacy and safety of safinamide (50–100 mg/day) as adjunctive treatment to levodopa in Chinese PD patients^13^, with 16-week treatment period. The study showed safinamide as levodopa adjunctive could significantly reduce the total daily OFF time by 1.10 h/day than placebo, and could improve ON time, Clinical Global Impression (CGI) scale score, Unified Parkinson’s Disease Rating Scale (UPDRS) score, and 39-Item Parkinson’s Disease Questionnaire (PDQ-39) score. In addition, there was no significant difference found in safety between safinamide and placebo in this study. Two multicenter, randomized, double-blind, placebo-controlled clinical trials evaluated the efficacy and safety of rasagiline as a levodopa adjunctive drug in Chinese PD patients (Zhang^14^ (NCT01479530) and Zhang^15^, hereinafter referred as Study 1 and Study 2). Study 1 divided patients randomly into rasagiline group (1 mg/day) and placebo group for 16 weeks, and found combination treatment of rasagiline could significantly reduce the total daily OFF time by 0.5 h/day than placebo, and also could improve the scores of CGI scale, UPDRS, and PDQ-39 scale. Study 2 divided patients randomly into rasagiline group (1 mg/day) and placebo group for 12 weeks, andfound significant decrease of total mean daily OFF time and increase of UPDRS score in rasagiline as adjunctive to levodopa group. Both studies didn’t find the significant difference in the safety between rasagiline and placebo.

### Study selection and feasibility assessment

IPD was derived from XINDI^13^, in which 350 patients received 100 mg/day of safinamide (safinamide increased from 50 mg to 100 mg/day after 14 days) or placebo for 16 weeks. AgD was derived from Study 1^14^, in which 310 patients received 1 mg/day rasagiline or placebo for 16 weeks. Study 1 was selected as the comparator trial because it has the same treatment period as XINDI, while Study 2 has a shorter treatment period (12 weeks).

This study evaluated the feasibility of MAIC between XINDI and Study 1 in terms of study design, inclusion criteria, treatment groups, outcomes, etc^14^. Patients enrolled in XINDI were diagnosed with idiopathic PD for more than 3 years and had been receiving a stable dose of levodopa, with a daily OFF-time ≥ 1.5 h. Patients enrolled in Study 1 were diagnosed with idiopathic PD and had been receiving a stable dose of levodopa, with a daily OFF time ≥ 1 h. An anchored MAIC was considered to be feasible based on the common comparator of placebo group in both RCTs.

### Indirect treatment comparisons

Matching-adjusted indirect comparison (MAIC) is a validated method for comparing the relative efficacy and safety of two treatments from different studies when individual patient data (IPD) is available^16^. MAIC usually compares IPD from the trial of interest and aggregate data (AgD) from the comparator trial. To improve comparability and minimize the difference in patient characteristics between IPD and AgD, researchers will weight IPD data by the relevant patient characteristics using methods such as propensity matching or regression adjustment^17^. MAIC could be classified as anchored or unanchored MAIC based on whether there is a common comparator (i.e. placebo arm)^18^, and the anchored MAIC is often preferred since it is less biased^18^.

An anchored MAIC was performed as the primary analysis in this study, the goal is to compare two treatments indirectly by adjusting for differences in baseline characteristics of patients from different trials. The MAIC approach in this study used IPD from the XINDI study and adjusted the study population to match the average baseline characteristics reported for Study 1. Prior to matching, unadjusted baseline characteristics were compared between the two samples (i.e., the active treatment and placebo arms) using Wald tests for continuous variables and chi-square tests (or the Fisher exact test for expected counts < 5) for categorical variables. Baseline characteristics that significantly differed between studies or served as clinically meaningful effect modifiers were matched to account for possible differences at baseline across the XINDI study and Study 1.

To balance the relevant patient characteristics between IPD (from XINDI study) and AgD (from Study 1), IPD was matched to AgD by assigning weights. Individual patients in the XINDI study were assigned weights such that weighted means and proportions for baseline characteristics in the pooled sample exactly matched those reported for the Study 1 population. Weights were derived using a logistic regression model estimating the propensity of a patient to enroll in the XINDI study, with the following baseline characteristics as covariates: age, sex, body mass index (BMI), and OFF time at baseline, which represented the patients’ demographics and PD progression.

After matching baseline demographics and clinical characteristics, we got a weighted XINDI dataset. The statistical analysis of MAIC in this study was performed in this weighted XINDI dataset. Then the effective sample size (ESS) was calculated, which is a crucial metric that reflects the actual information content of the weighted sample after adjusting for baseline differences in MAIC study. It is different from the actual number of patients in the study because it accounts for the variability and distribution of the weights. The ESS typically decreases after applying weights, indicating the true number of independent observations that contribute to the analysis.

This study compared the common clinical outcomes which XINDI and Study 1 both reported. The primary efficacy outcome was the change in mean total daily OFF time from baseline to week 16, which was evaluated by the patient diary. Secondary efficacy outcomes were the changes in the mean of UPDRS part III (motor) scores, PDQ-39 summary score and PDQ-39-dimension scores of activities of daily living, emotional well-being, mobility, and stigma. This study also evaluated the safety outcomes including the frequency of adverse events (AEs), serious adverse events (SAEs), and discontinuations due to adverse events (DCAEs).

The matching-adjusted indirect comparison method employed in this study adheres to the guidelines outlined in the NICE DSU Technical Support Document 18, published by the University of Sheffield^19^.

### Statistical analysis

Baseline demographics and clinical characteristics were compared between before and after matching, with Wald test for continuous data and chi-square test for categorical data (Fisher’s exact test for data with small frequency). In primary outcome analysis, study center and the treatment were set as independent factors and the baseline OFF time was set as the covariate. Mean change in total daily OFF-time was analyzed by a weighted covariance model to obtain the least-squares mean (LSM) with 95% confidence interval (95% CI) for treatment difference. The secondary efficacy outcomes were analyzed using the weighted covariance models as well. Safety outcomes (including rates of AEs, SAEs, and DCAEs) were calculated based on weighted XINDI dataset. Bucher method^20^ was used in primary and secondary outcome analysis to generate the mean difference (MD) of efficacy outcomes and odds ratio (OR) of safety outcomes, each with a 95% CI. All statistical analysis was performed in R software (version 4.2.3).

### Ethical compliance statement

This study was a post hoc analysis of the XINDI study and its ethical compliance was approved by the Ethics Committee of Shanghai Ruijin Hospital. Informed patient consent was not necessary for this post hoc analysis. All study participants recruited by the XINDI study provided informed consent. Furthermore, it was confirmed that The XINDI research involving human participants was conducted in accordance with the Declaration of Helsinki.

## Results

### Patient baseline demographics and clinical characteristics

There were 305 and 301 patients included in the original XINDI and Study 1, respectively. In a comparative analysis of the unadjusted baseline demographics and clinical characteristics between the XINDI study and Study 1, statistically significant differences were observed, including male proportion (58.0% vs. 66.0%, *p* = 0.003), BMI (mean 23.9 vs. 23.1, *p* < 0.001), total daily ON time (mean 10.2 vs. 9.4 h, *p* < 0.001), UPDRS part II score during ON time (mean 12.0 vs. 7.1 points, *p* < 0.001), and UPDRS part III score during ON time (mean 27.1 vs. 24.7 points, *p* = 0.001). After MAIC matching, weights were calculated and applied to adjust for differences in baseline characteristics between two study populations. The ESS was 271 in the weighted XINDI dataset, including 135 in the safinamide group and 136 in the placebo group. The decrease of the ESS was due to the nature of the weighting process, which adjusted the influence of individual data points to better match the target population’s baseline characteristics. The above baseline demographics and clinical characteristics were balanced after matching (*p* > 0.05), except for the significant difference in UPDRS part II score (mean 12.2 vs. 7.1 points, *p* < 0.001). Baseline demographics and clinical characteristics before and after matching are shown in Tables 1 and 2, respectively.

**Table 1:** Baseline demographics and clinical characteristics before matching.

	Safinamide			Rasagiline		
	Safinamide (*N* = 151)	Placebo (*N* = 154)	Overall (*N* = 305)	Rasagiline (*N* = 163)	Placebo (*N* = 158)	Overall (*N* = 321)
Age [years, mean (SD)]	61.4 (9.3)	61.8 (9.3)	61.6 (9.3)	62.7 (8.9)	61.7 (9.9)	62.2(9.4)
Male (%)^**^	60.9	55.2	58.0	63.0	69.0	66.0
BMI [kg/m^2^, mean (SD)]^**^	23.9 (3.0)	23.9 (3.1)	23.9 (3.0)	23.1 (3.2)	23.2 (3.3)	23.1 (3.2)
Overweight (%)	4.6	4.6	4.6	–	–	–
Total daily off-time [h, mean (SD)] ^a^	5.9 (2.8)	5.6 (3.1)	5.7 (3.0)	6.1 (2.6)	6.1 (2.7)	6.1 (2.6)
Total daily on-time [h, mean (SD)] ^a**^	10.1 (2.8)	10.3 (3.1)	10.2 (3.0)	9.4 (2.4)	9.3 (2.5)	9.3(2.4)
UPDRS part II score-on phase [mean (SD)] ^b**^	12.0 (5.2)	12.0 (5.9)	12.0 (5.6)	6.8 (4.6)	7.3 (5.1)	7.1 (4.9)
UPDRS part III score-on phase [mean (SD)] ^b**^	27.4 (12.3)	26.8 (13.5)	27.1 (12.9)	23.8 (10.5)	25.6 (10.5)	24.7 (10.5)

^a^ *n* = 150 for the placebo group and *n* = 158 for the rasagiline group in the rasagiline study.

^b^ *n* = 152 for the placebo group and *n* = 158 for the rasagiline group in the rasagiline study.

the pooled Safinamide group vs. pooled Rasagiline group *p < 0.05, **p < 0.01.

*ESS* effective sample size, *SD* standard deviation, *BMI* body mass index.

**Table 2:** Baseline demographics and clinical characteristics after matching.

	Safinamide			Rasagiline		
	Safinamide (ESS = 135)	Placebo (ESS = 136)	Overall (*N* = 271)	Rasagiline (*N* = 163)	Placebo (*N* = 158)	Overall (*N* = 321)
Age [years, mean (SD)]	62.1 (9.4)	62.3 (9.1)	62.2 (9.2)	62.7 (8.9)	61.7 (9.9)	62.2(9.4)
Male (%)	67.7	64.3	66.0	63.0	69.0	66.0
BMI [kg/m^2^, mean (SD)]	23.1 (3.0)	23.2 (3.0)	23.2 (3.0)	23.1 (3.2)	23.2 (3.3)	23.1 (3.2)
Overweight (%)	4.5	4.2	4.4	–	–	–
Total daily off-time [h, mean (SD)] ^a^	6.2 (2.9)	6.0 (3.2)	6.1 (3.1)	6.1 (2.6)	6.1 (2.7)	6.1 (2.6)
Total daily on-time [h, mean (SD)] ^a^	9.9 (2.9)	10.1 (3.0)	10.0 (2.9)	9.4 (2.4)	9.3 (2.5)	9.3(2.4)
UPDRS part II score-on phase [mean (SD)] ^b **^	12.1 (4.9)	12.2 (5.9)	12.2 (5.4)	6.8 (4.6)	7.3 (5.1)	7.1 (4.9)
UPDRS part III score-on phase [mean (SD)] ^b^	28.0 (12.6)	27.6 (13.8)	27.8 (13.2)	23.8 (10.5)	25.6 (10.5)	24.7 (10.5)

^a^ *n* = 150 for the placebo group and *n* = 158 for the rasagiline group in the rasagiline study.

^b^ *n* = 152 for the placebo group and *n* = 158 for the rasagiline group in the rasagiline study.

*p < 0.05, **p < 0.01.

*ESS* effective sample size, *SD* standard deviation, *BMI* body mass index.

### Efficacy outcomes

In the MAIC analysis, it was observed that treatment with safinamide at a dosage of 100 mg/day resulted in a statistically significant reduction in the mean total daily OFF time. Specifically, from baseline to week 16, there was a least square mean (LSM) difference of 1.2 h/day, indicating a reduction of 0.7 h/day (mean difference [MD] = − 0.7, 95% confidence interval [CI] − 1.40 to − 0.02) when compared to rasagiline at a dosage of 1 mg/day, which showed a reduction of 0.5 h/day. This suggests a superior efficacy of safinamide in managing OFF time in patients.

Safinamide was also found to be more effective than rasagiline in improving motor functions, as indicated by the reduction in the UPDRS-III scores. Patients treated with safinamide experienced a mean reduction of 4.5 points, whereas those treated with rasagiline experienced a mean reduction of 1.6 points (MD = − 2.9, 95% CI − 5.28 to − 0.52) from baseline to week 16, demonstrating a clear advantage for safinamide in this measure.

However, the changes in several other health-related quality of life measures did not show significant differences between the two treatments. The PDQ-39 summary index showed a mean difference of -2.2 points (95% CI − 5.26 to 0.84) between safinamide and rasagiline from baseline to week 16, indicating no significant advantage. Similarly, the activities of daily living score (MD = − 0.6, 95% CI − 5.44 to 4.24), emotional well-being score (MD = − 3.2, 95% CI − 8.36 to 1.90), mobility score (MD = -3.5, 95% CI − 7.84 to 0.84), and stigma score (MD = − 3.9, 95% CI − 9.27 to 1.57) did not show significant differences from baseline to week 16, although the safinamide group exhibited higher numerical values.

The detailed results of this MAIC analysis are comprehensively presented in Table 3, providing further insights into the comparative efficacy and safety profiles of the two treatments.

**Table 3:** MAIC of efficacy and safety for safinamide versus rasagiline.

	Safinamide vs. placebo	Rasagiline vs. placebo	Measure	Safinamide vs. rasagiline
	LSM (SE)	LSM (SE)		
Efficacy outcomes				
Off-time	− 1.2 (0.28) **	− 0.5 (0.22) *	MD (95% CI)	− 0.7 (− 1.40, − 0.02) *
UPDRS III (on phase)	− 4.5 (0.96) **	− 1.6 (0.74) *	MD (95% CI)	− 2.9 (− 5.28, − 0.52) *
PDQ-39 summary index score	− 4.0 (1.08) **	− 1.8 (1.12)	MD (95% CI)	− 2.2 (− 5.26, 0.84)
Activities of daily living score	− 6.7 (1.74) **	− 6.1 (1.76) **	MD (95% CI)	− 0.6 (− 5.44, 4.24)
Emotional well-being score	− 5.6 (1.69) **	− 2.4 (2.00)	MD (95% CI)	− 3.2 (− 8.36, 1.90)
Mobility score	− 6.0 (1.57) **	− 2.5 (1.56)	MD (95% CI)	− 3.5 (− 7.84, 0.84)
Stigma score	− 6.4 (1.99) **	− 2.5 (1.92)	MD (95% CI)	− 3.9 (− 9.27, 1.57)
Safety outcomes				
AEs	1.9 (0.26)	1.1 (0.23)	OR (95% CI)	1.6 (0.83, 3.19)
SAEs	1.5 (0.62)	1.4 (0.60)	OR (95% CI)	1.1 (0.21, 6.14)
Discontinuations due to AEs	0.8 (0.52)	1.2 (0.62)	OR (95% CI)	0.7 (0.14, 3.28)

*p < 0.05, **p < 0.01.

*LSM* least-squares mean, *SE* standard error, *MD* mean difference, *UPDRS* unified Parkinsons’ disease rating scale, *PDQ-39* 39-Item Parkinson’s Disease Questionnaire, *AEs* adverse events, *SAEs* serious adverse events, *MAIC* matching-adjusted indirect comparison, *MD* mean difference, *OR* odds ratio, *CI* confidence interval.

### Safety outcomes

There was no significant difference in safety between safinamide and rasagiline in MAIC analysis, including AEs (OR = 1.6, 95% CI 0.83–3.19), SAEs (OR = 1.1, 95% CI 0.21–6.14), and DCAEs (OR = 0.7, 95% CI 0.14–3.28). Safety results are shown in Table 3.

## Discussion

This paper presents a comparative analysis of two MAO-BIs (safinamide and rasagiline) that provide important evidence to support Health Technology Assessments (HTAs) and inform public health decision-making. Researchers employed an anchored MAIC method, utilizing the placebo arm as a common comparator to evaluate safinamide versus rasagiline in Chinese PD patients experiencing motor fluctuations. After adjusting for key demographic and clinical characteristics (age, sex, BMI, and baseline OFF time), results indicated that that safinamide could significantly reduce OFF time by 0.7 h per day and decrease the UPDRS-III score by 2.9 points, respectively, when compared to rasagiline. The safinamide group exhibited better PDQ-39 scores, indicating a trend towards superior efficacy. In addition, safinamide is similarly safe and well-tolerated as rasagiline.

The safety results of this paper are consistent with previous studies that have reported. Conversely, in efficacy outcomes, the network meta-analysis study, which included 31 randomized controlled trials, reported no significant differences between safinamide and rasagiline in efficacy outcomes^5,21^. This discrepancy may be attributed to the differences between the traditional meta-analysis and MAIC statistical methods^22^. Traditional meta-analysis has the advantage of being a well-established and widely used statistical method. It allows for the aggregation of results from multiple independent studies, thereby increasing the overall sample size and statistical power, which in turn leads to more precise estimates of treatment effects^23^. Meta-analysis is particularly effective when comparing studies that are similar in design, patient characteristics, and interventions, as it can combine the results to provide a comprehensive evaluation of the overall efficacy and safety of treatments^23^. However, meta-analysis may be limited by its assumption of homogeneity across studies. If the included studies differ significantly in terms of population characteristics, interventions, or outcome measures, the results may be biased^23^. In contrast, one of the main advantages of MAIC is its ability to control for baseline characteristic differences between studies. By adjusting for key demographic and clinical characteristics (such as age, sex, weight, and baseline disease severity), MAIC minimizes the impact of these differences on treatment outcome comparisons, thereby providing more reliable indirect comparison results. In this study, researchers use MAIC analysis to adjusts for these differences by accounting for key demographic and clinical characteristics, thus reducing potential bias. In the MAIC analysis conducted in this study, significant improvements were observed in both primary and secondary efficacy outcomes after adjusting the baseline characteristics (covariates): age, sex, BMI, and baseline OFF time. After matching, there were no differences in the following baseline characteristics between the XINDI study and Study 1 groups, including age, sex, BMI, and OFF time at baseline. And then, for the XINDI study dataset, there were no significant differences in baseline characteristics for age and baseline OFF time after matching compared to pre-matching. However, after matching, the proportion of males increased, and the proportion of individuals who were overweight (BMI > 30) decreased. A post-hoc analysis from the XINDI study^24^ indicated that female patients receiving treatment with safinamide showed better therapeutic outcomes compared to males, although these differences did not reach statistical significance. However, despite the increased male proportion in our study, the efficacy outcomes still showed significant improvement.

The efficacy difference between safinamide and rasagiline groups, despite similar baseline demographics and clinical characteristics, may be attributed to the unique action mechanism of safinamide. Safinamide’s efficacy involves inhibiting specific ion channels and reducing pathologically elevated glutamate releas^25^, providing improved efficacy compared to traditional MAO-B inhibitors such as rasagiline. In addition to the mechanism, racial difference may be another reason for divergent conclusions from meta-analyses^5,21^. According to a post- hoc analysis of the SETTLE study^26^, it was found that safinamide could improve motor functions significantly in Asians, but not in the Caucasian population. This could be interpreted by the lower average body weight in Asians. In case of identical dosages of medications, the blood concentrations are higher in Asians with lower body weights, which may contribute to better efficacy^26^.

According to the MDS treatment guidelines, safinamide is primarily recommended for use in mid-to-late-stage Parkinson’s disease patients with poorly controlled motor fluctuations^27^. However, no significant advantage was observed in non-motor symptoms such as total PDQ-39 scores, activities of daily living, and emotional well-being, despite safinamide showing more favorable scores in these areas. A study involving 45 patients with sleep disturbances, assessed using the Parkinson’s Disease Sleep Scale-2 (PDSS-2), demonstrated that safinamide exhibited a greater improvement in both subjective sleep quality and objective sleep structure compared to rasagiline^28^. Compared to patients treated with rasagiline, a higher proportion of patients on safinamide transitioned to the “good sleeper” category^28^, which may be attributed to its dual mechanism of action, which includes both dopaminergic inhibition and regulation of glutamate release^29^. Although safinamide shows potential in improving non-motor symptoms in PD patients^29^, the optimal therapeutic approach for non-motor symptoms still requires further clinical research for validation.

The dual mechanism of action of safinamide, involving calcium channel blockade and glutamate modulation, highlights its potential application in the early stages of Parkinson’s disease^30^. Glutamate, the primary excitatory neurotransmitter in the central nervous system, is closely linked to motor symptoms in Parkinson’s disease due to its excessive release^31^. By inhibiting glutamate release, safinamide may help protect neurons from excitotoxic damage, potentially reducing the occurrence and progression of motor complications^31^. However, clinical trial evidence regarding the use of safinamide in early Parkinson’s disease patients without motor fluctuations is currently lacking. Future research should be encouraged to explore the application of safinamide in early-stage Parkinson’s disease to assess its impact on motor complications, thereby further enriching the evidence base.

A major strength of this study is the application of MAIC, which allows for a more accurate comparison by adjusting for baseline characteristics of the populations. This method helps to mitigate the biases inherent in indirect comparisons and provides a robust analysis of the therapeutic benefits and risks. However, there are several limitations to these analyses. Firstly, this study only compared the short-term efficacy (16 weeks) between safinamide and rasagiline. Secondly, Other efficacy outcomes (such as on-time, UPDRS-II, CGI-I, etc.) which may be also of significance in clinical practice could not be compared, as they were not reported in both studies included. Thirdly, due to the fact that the study population consists entirely of Asians, the results reflect the situation regarding the use of MAO-B inhibitors in combination with levodopa for treating motor fluctuations in Parkinson’s disease patients within this ethnic group, and therefore, the findings may not be generalizable to other ethnic populations. Forth, not to further match baseline data to adjust for statistical differences in UPDRS Part 2, is a limitation of this study. The IPD-sourced study only measured UPDRS Part 2 scores during on-time period, while the AgD-sourced study measured both on-time and off-time UPDRS Part 2 score, potentially leading to discrepancies in measurement. As Additional adjustments to baseline scores could have led to a reduction in the effective sample size (ESS), potentially affecting the accuracy and precision of the results. Finally, this study was performed based on RCTs, whose conditions differed from the real clinical settings, as the subjects included were those who met certain criteria.

The findings of this study have important implications for clinical practice. The superior efficacy of safinamide suggests that it could be recommended as a preferred option for China patients with PD. Future research should focus on investigate the long-term efficacy of safinamide in Chinese PD patients to further validate our findings. Additionally, Head-to-head clinical trials comprise between safinamide and rasagiline could provide deeper insights into optimizing the use of MAO-BIs inhibitors in clinical practice.

## Conclusion

Compared to rasagiline 1 mg/day, safinamide 100 mg/day as adjunctive treatment to levodopa could significantly improve motor fluctuations for PD patients in China, without compromising safety.

## Data Availability

Raw data relevant to the conclusions of this study will be made available by the corresponding authors upon reasonable request. Requests for data sharing should include a clear purpose and justification, and access will be granted in compliance with applicable ethical guidelines and data-sharing policies to ensure the protection of participant confidentiality and intellectual property.
